# Experimental Studies on the Therapeutic Potential of *Vaccinium* Berries in Breast Cancer—A Review

**DOI:** 10.3390/plants13020153

**Published:** 2024-01-05

**Authors:** Naser A. Alsharairi

**Affiliations:** Heart, Mind and Body Research Group, Griffith University, Gold Coast, QLD 4222, Australia; naser.alsharairi@gmail.com

**Keywords:** *Vaccinium* spp., fleshy berry fruits, bioactive compounds, breast cancer, experimental models

## Abstract

Breast cancer (BC) is the largest contributor to cancer deaths in women worldwide. Various parts of plants, including fruits, are known for their therapeutic properties and are used in traditional medicine. Fruit species exhibit anticancer activities due to the presence of bioactive natural compounds such as flavonoids and carotenoids. The *Vaccinium* spp. are fleshy berry-like drupes and are rich in bioactive compounds, with flavonols, flavanols, chalcones, and phenolic acids as the major groups of compounds. While there is clear evidence linking *Vaccinium* berries with a decreased risk of BC both in in vivo and in vitro experiments, the exact mechanisms involved in the protective effects of *Vaccinium* spp. rich extracts on BC cells are not fully understood. Thus, the purpose of this review is to highlight the mechanisms of action involved in the therapeutic potential of *Vaccinium* berries against BC in experimental models.

## 1. Introduction

Breast cancer (BC) is regarded as the most common cancer in women globally, with an estimated 2.3 million cases and close to 700,000 deaths occurring in 2020 [1]. The burden of BC is projected to reach 3 million cases and 1 million deaths by 2040 [2]. China and South Korea had relatively low BC incidence, but showed higher mortality trends than the USA, Australia, and the UK during 2015–2020 [1]. The etiology of BC is related to many non-modifiable and modifiable risk factors. The non-modifiable factors include older age, menstrual period/menopause, pregnancy/breastfeeding, previous history of BC/radiation therapy, and non-cancerous breast diseases [3]. Smoking, alcohol intake, low physical activity, a high body mass index, hormonal replacement therapy, insufficient vitamin supplementation, ultra-processed food intake, and exposure to chemicals/artificial light also play a role in BC as modifiable risk factors [3,4]. BC is classified into four major molecular subtypes: luminal A, luminal B, human epidermal growth factor receptor-2 (HER2)-positive, and basal-like/triple-negative (TNBC) [3,5]. Luminal A and B comprise 60% and 10% of all BC cases, respectively, and express estrogen (ER) and progesterone (PR) receptors [3,5]. The luminal A subtype, distinguished by ER+/PR+/HER2-, has lower proliferation (evaluated by Ki67 antigen expression) and better prognosis than the luminal B subtype [3,5,6]. The HER2-positive subtype, a member of the receptor tyrosine kinase family, represents about 10% of BC cases, and is characterized by the absence of ER/PR, higher HER2 expression, and is more aggressive and faster-growing than luminal cancers [3,5,7]. The TNBC subtype accounts for 20% of BC cases, and is characterized by ER-/PR-/HER2-, high expression of proliferation-related genes, an aggressive phenotype, and early relapse [3,5,8]. TNBC is further classified into six subtypes: mesenchymal (M), basal-like 1 (BL-1), basal-like 2 (BL-2), mesenchymal stem-like (MSL), luminal androgen receptor (LAR), and immunomodulatory (IM), which feature a high expression of genes associated with growth factor/cytokine signaling, cell motility, and cell differentiation/natural killer cell pathways [8].

Several preclinical and clinical trials have evaluated evidence-based treatment for BC. Neoadjuvant endocrine therapy (NET) either alone or in combination with targeted agents, such as cyclin-dependent kinase 4/6 (CDK 4/6), mammalian target of rapamycin (mTOR), and phosphatidylinositol-3 kinase (PI3K) inhibitors, has clinical benefit for patients with luminal BC [3,9,10]. Trastuzumab, tyrosine kinase inhibitors (lapatinib, afatinib, pyrotinib), P13K/serine, threonine kinase/mTOR (PI3K/AKT/mTOR), and blocking drugs (e.g., trastuzumab, everolimus, paclitaxel) are regarded as options for the treatment of HER2-positive BC [11,12]. The common treatment options of TNBC are Poly (ADP-ribose) polymerase (PARP) inhibitors (Talazoparib, Rucaparib, Niraparib, Olaparib), growth factor inhibitors (Axitinib, Afatinib, Nazartinib, Pazopanib, Bevacizumab), mTOR inhibitors (everolimus, RapaLink-1, rapamycin), PI3K inhibitors (Idelalisib), immune checkpoints (Ipilimumab, Nivolumab), and mitogen-activated protein kinase (MAPK) inhibitors (Trametinib, Dabrafenib) [13].

Fruits are categorized as fleshy or dry fruits, depending on their water content at ripening [14]. Fleshy-fruited species contain high water contents, grow in relatively low-elevation forests, and prefer evapotranspiration-shaded habitats, where plants are exposed to low wind speeds and direct sunlight, and thus attract frugivores who eat the fruits and disperse the indigestible seeds [14]. Fleshy fruits are further categorized as climacteric (e.g., banana, apple, mango, tomato) or non-climacteric (e.g., strawberry, grape, berry) based on their ethylene biosynthesis during ripening [15]. Ethylene production is increased in climacteric fruits at the onset of ripening, whereas abscisic acid (ABA) production is increased in non-climacteric fruits [16,17]. Non-climacteric fruits are also sensitive to low levels of ethylene [17,18,19]. In climacteric fleshy fruit ripening, ethylene plays a key hormonal role in stimulating the differential expression of many gene encoding transcription factors that regulate starch/pigments and carotenoid accumulation, cell wall softening, texture change and aroma, flavor, and skin color development [15,18]. Ripening is considered an important stage where many bioactive flavonoids are accumulated in fleshy fruits [19].

*Vaccinium* berries are considered non-climacteric fleshy fruits. The genus *Vaccinium* is the largest polyphyletic member of the Ericaceae family, which consists of several fruit-bearing species that generate fleshy fruits classified as berries [20]. *Vaccinium* spp. such as *V. corymbosum* L./*angustifolium* L. (blueberry), *V. myrtillus* L. (bilberry), *V. uliginosum* L. (bog bilberry), *Arctostapylos uva-ursi* L. (bearberry), *V. vitis-idaea* L. (lingonberry), and *V. macrocarpon* L./*oxycoccos* L. (cranberry) have been widely used as medicinal plants in Europe and Central/North America, due to the high levels of bioactive compounds present in their parts, which exert strong anti-inflammatory and antioxidant effects against some diseases, such as neurodegenerative disorders, atherosclerosis, diabetes, and cancer, as demonstrated by in vitro and in vivo studies [21,22,23,24,25,26]. *Vaccinium* berries were also used for treating several conditions, such as gastrointestinal disorders, respiratory system infections, hepatitis, and renal/kidney stones [25]. The types and levels of natural flavonoids vary in *Vaccinium* berries depending on their species, latitude, geographical origin, cultivation conditions, and ripeness stage [21]. The main bioactive compounds identified in *Vaccinium* berries were flavonols (quercetin, isoquercitrin, rutin, kaempferol, myricetin, isorhamnetin, syringetin), flavanols (catechin, epicathechin, epigallocatechin, proanthocyanidins, anthocyanins), chalcones, phenolic acids, and stilbene-based derivatives (e.g., piceatannol, resveratrol, pterostilbene) [21,22,23,25,27,28,29,30].

The bioavailability of bioactives in *Vaccinium* berries is important for evaluating their beneficial effects as potential therapeutic agents in BC. Previous intervention studies showed increases in plasma quercetin levels up to 50% in volunteers consuming a diet containing lingonberries and bilberries for 6 weeks [31]. Research evidence points to increased plasma concentrations and urinary excretions of different polyphenols (quercetin, caffeic, protocatechuic, p-coumaric, and vanillic acid) in adults consuming products prepared from lingonberries, bilberries, chokeberries, and black currants [32]. Human research has revealed high absorption of anthocyanidin peonidin glycosides and chlorogenic acids in adults consuming wild blueberries [33]. The cranberry proanthocyanidin-A2 is detected in the plasma and urine of healthy adults in very high contents after being produced from polymers/oligomers through microbiota-mediated catabolism [34]. The stability of polyphenols and anthocyanins in wild blueberries (*V. angustifolium*) is generally high during simulated in vitro digestion [35]. The stability of anthocyanins during in vitro digestion showed that hydrophobic anthocyanins are easier to absorb than hydrophilic anthocyanins [36]. The administration of a blueberry–grape combination to mice increases plasma flavonols, phenolic acids, and resveratrol levels [37].

Despite recent reviews on *Vaccinium* berries (lingonberry, bilberry, blueberry, and cranberry) as potential therapeutic agents for colorectal, oral, skin, and lung cancers in vitro/in vivo experiments [38,39,40,41], to date there has been no review of the effects and mechanisms of *Vaccinium* berries and their bioactive compounds in BC treatment. Thus, this review aims to explore the mechanisms involved in the therapeutic potential of *Vaccinium* berries in BC.

## 2. Methods

A literature search was conducted using the PubMed/Medline database until 1 December 2023. The search focused on research examining the potential mechanisms of *Vaccinium* berries in BC treatment. Search terms included the following: “*Vaccinium*” OR “*Vaccinium* berries” OR “blueberry” OR “bilberry” OR “bog bilberry” OR “bearberry” OR “lingonberry” OR “cranberry” AND “BC”. All relevant papers were evaluated for inclusion by identifying both in vitro and in vivo experiments. Experiments focusing on the therapeutic effects of wild berries against BC cells were not considered. The search extracted 2439 articles based on the search terms, of which 32 articles were selected for inclusion.

## 3. Blueberry and Cranberry in BC Treatment

A few experiments have demonstrated the therapeutic efficacy of blueberry and cranberry extracts in BC cells. The ethanolic extracts from blueberry cultivars (Tifblue and Premier) showed an inhibitory effect on carcinogen benzo(a)pyrene-mutated BC in vitro [42]. In vitro and in vivo experiments revealed that blueberry inhibits genes involved in TNBC cell proliferation, migration, and motility, while upregulating genes involved in cell apoptosis via inhibiting the PI3K/Akt and nuclear factor kappa-B (NFκB) signaling pathways [43]. Intake of blueberry powder at a concentration of 5% inhibits TNBC cell proliferation/metastasis and influences anti-inflammatory cytokine production in mice via suppressing the Wnt/β-catenin signaling pathway. Further, blueberry powder at a concentration of 10% increases the apoptotic potential of TNBC cells [44]. Blueberry inhibits the metastasis and tumor growth of TNBC cells in Xenograft mice through increasing anti-inflammatory cytokine production [45]. The blueberry blend (Tifblue and Rubel) has shown anti-proliferative effects on 17β-estradiol (E2)-mediated BC in August-Copenhagen-Irish (ACI) rats by reducing ER-related gene expression in the mRNA/protein levels and E2-specific miRNAs [46]. Treatment with blueberry for an average of 24 weeks inhibits estrogen-induced mammary tumorigenesis in ACI rats by reducing the expression of E2-metabolizing enzymes [47].

Mice treated with either non-fermented blueberry juice (NBJ) or polyphenol-enriched blueberry preparation (PEBP) at different concentrations showed a significant inhibition of proliferation, tumor growth, metastasis, invasion, mobility, and mammosphere formation in BC cells. This was mediated by modulating the cellular signaling cascades of breast mammary cancer stem cells (CSCs), including the inhibition of the signal transducer and activator of transcription 3 (STAT3), extracellular-signal regulated kinase 1/2 (ERK 1/2), PI3K, and Akt signaling pathways, while activating the signaling pathways of mitogen-activated protein kinase (MAPK), c-Jun N-terminal kinase (JNK), and stress-activated protein kinase (SAPK) [48]. In vitro experiments reported a significant BC cell invasion inhibition when treated with NBJ and/or PEBP at different concentrations. This was observed through downregulating the expression of oncogenic micro (miR)-210 and neuroblastoma RAS viral oncogene homolog (NRAS), while upregulating the expression of tumor suppressor miR-145 and the forkhead box O1 (FOXO1) transcription factor [49]. Only one study showed that blueberry and cranberry suppress the proliferation of BC cells. This effect was accompanied by arresting the cell cycle at the G_1_ phase through downregulating cell cycle-related gene expression [50]. 

Table 1 highlights the therapeutic potential of blueberry and cranberry in in vitro and/or in vivo models of BC.

## 4. Natural Bioactive Compounds Derived from *Vaccinium* Berries in BC Treatment

This section presents the bioactive compounds derived from *Vaccinium* berries and the mechanisms of their therapeutic potential in BC. 

### 4.1. Flavanols and Phenolic Acids

Several experiments so far showed effective results of flavanols and phenolic acids, from the most-occurring phytochemicals in blueberry, cranberry, and bilberry, in BC treatment. In vitro experiments have shown that flavanols (anthocyanins, proanthocyanidins, and catechins) extracted from cranberry press cake inhibit BC cell proliferation via the induction of cell cycle arrest in phases G_1_ and G_2_/M, leading to apoptosis [51]. The treatment of BC cells with cranberry phytochemical extracts (cyanidin, catechins, and gallic acid) demonstrated significant inhibition of proliferation, as well as the induction of apoptosis and cell cycle arrest in phases G_0_/G_1_ and G_1_/S in vitro [52]. Treatment with cranberry- and blueberry-derived anthocyanins inhibits BC cell proliferation at high concentrations (150 and 200 μg/mL) in vitro [53]. 

Blueberry anthocyanins and anthocyanin-pyruvic acid adduct extracts inhibit BC cell proliferation and invasion at a concentration of 250 μg/mL in vitro. Moreover, the anthocyanin-pyruvic acid adduct extract showed apoptotic activities in MCF-7 cells at the same concentration by increasing the activity of caspase-3 [54]. Blueberry anthocyanin extracts exert anti-proliferative effects, also in vivo, by downregulating the expression levels of cytochrome P4501A1 (CYP1A1) in BC cells [55]. Anthocyanins extracted from gardenblue blueberry in combination with the chemotherapeutic drugs cisplatin (30.45 μg/mL) and doxorubicin (6.97 μg/mL) showed anti-proliferative effects in BC cells by inducing apoptosis through decreasing DNA damage [56]. 

In vivo experiments in mice showed that treatment with a polyphenolic mixture derived from fermented blueberry juice and containing gallic acid, catechol, and protocatechuic acid resulted in the suppression of mammosphere formation in BC cells by increasing the expression of miR-145 and FOXO1 [57]. Blueberry phenolic acids, and hippuric acid in particular, have exerted in vivo inhibitory effects on mammosphere formation in MDA-MD-231 cells and their CD441/CD242/ESA1 subpopulation through the induction of the tumor suppressor phosphatase and tensin homologue deleted on chromosome ten (PTEN) expression [58].

Bilberry anthocyanin extract was shown to inhibit proliferation and induce apoptosis and G_2_/M-phase cell cycle arrest in MCF7-GFP tubulin cells at high concentrations (≥0.5 mg/mL) in vitro [59]. Anthocyanidin aglycone (Anthos) isolated from the standardized anthocyanin-enriched extract of bilberry has demonstrated antiproliferative and anti-inflammatory activities via the inhibition of TNFα-induced NF-κB levels in vitro and in vivo, with no toxic side effects observed against BC cells [60]. In another in vitro and in vivo study, bilberry Anthos inhibited the proliferation, viability, migration, invasion, and metastasis of BC cells through the induction of apoptosis and cell cycle arrest in phases G_0_/G_1_ and G_2_/M. The mechanisms underlying the effect are related to the modulation of epithelial-to-mesenchymal transition (EMT) markers and apoptosis-related proteins. Further, Anthos in combination with the paclitaxel drug inhibits tumor growth and metastasis in BC cells through the suppression of NF-kB activity [61]. Flavanol- and phenolic acid-rich extracts of the bilberry showed antimicrobial effects in BC cells in vitro through inhibiting the growth of *Escherichia coli* (*E. coli*) and *Salmonella typhimurium* (*S. typhimurium*) strains [62]. 

Experimental models that have highlighted the therapeutic potential of flavanols and phenolic acids in BC are summarized in Table 2.

### 4.2. Stilbene-Based Derivatives

Stilbene-based derivatives, including pterostilbene, piceatannol, and resveratrol, isolated from *Vaccinium* berries (blueberry, cranberry, bilberry, and lingonberry) have been demonstrated to have anti-BC effects. In vitro experiments using BC cells showed an inhibition of viability, and an induction of apoptosis and S-phase arrest after treatment with pterostilbene via increasing mitochondrial depolarization, superoxide anion, and caspase-dependent apoptosis in the cell cycle [63]. Pterostilbene in combination with tamoxifen, a nonsteroidal antiestrogen, showed anti-viability and apoptotic activities in BC cells at different concentrations in vitro [64]. Pterostilbene exerts proliferation, invasion, and viability inhibitory effects on BC cells in vitro, as indicated by decreasing heregulin-β1 (HRG-β1)-mediated matrix metalloproteinase-9 (MMP-9) expression through the suppression of the PI3K/Akt and p38 kinase signaling pathways [65]. Pterostilbene treatment was shown to suppress the in vitro and in vivo proliferation activities of BC cells by inducing apoptosis through the inhibition of the expression of ER-α36 and the MAPK/ERK and PI3K/Akt signaling pathways [66]. 

Pterostilbene enhances the in vitro apoptosis activities in tumor necrosis factor-related apoptosis-induced ligand (TRAIL)-resistant TNBC cells through the activation of the reactive oxygen species (ROS)-mediated p38/C/EBP-homologous protein (CHOP) signaling pathway, leading to death receptors and pro-apoptotic gene expression [67]. Pterostilbene has been reported in vitro and in vivo to inhibit tumor-associated macrophage (TAM)-induced invasive/metastatic potential and cancer stem cell (CSC) generation through the suppression of the NFκB signaling pathway and EMT-related molecules [68]. Pterostilbene showed strong antiproliferative activities and an induction of apoptosis and G_0_/G_1_-phase arrest both in vitro and in vivo. These effects occur in BC cells through the activation of pro-apoptotic molecules and the inhibition of signaling/anti-apoptotic molecules [69]. Treatment with pterostilbene suppresses proliferation, along with the stimulation of apoptosis and cell cycle arrest in phases G_1_ and G_2_/M in vitro. These effects are triggered by downregulating human telomerase reverse transcriptase (hTERT) through inhibiting cMyc expression and reducing telomerase levels in BC cells [70]. 

Piceatannol was reported to induce anti-migration, anti-invasion, and anti-adhesion activities in vitro by inhibiting MMP-9 expression and the PI3K/AKT/NF-κB signaling pathway in BC cells [71]. Treatment with resveratrol in combination with paclitaxel both in vitro and in vivo showed an inhibition of viability, along with the stimulation of apoptosis and S-phase cell cycle arrest in BC cells. This is mediated through reducing the accumulation of intracellular ROS and the expression of anti-apoptotic molecules [72]. The treatment of BC cells with resveratrol resulted in the inhibition of cell viability and the induction of cell apoptosis and G_1_-phase cell cycle arrest in vitro. This is triggered by the inhibition of the PI3K/Akt/mTOR signaling pathway, fatty acid synthase (FASN), cyclin D1, Akt phosphorylation, and the activation of PTEN and polyomavirus enhancer activator 3 (PEA3) expression [73]. 

Table 3 summarizes the results from experimental models that have highlighted the therapeutic potential of stilbene-based derivatives in BC. 

## 5. Limitations

Based on these findings, *Vaccinium* berries and their bioactives exert a wide range of therapeutic effects against BC cells, but these effects have not been investigated in clinical trials. Although the majority of experiments reported a range of therapeutic effects of stilbene-based derivatives and some phenolic acids against BC cells, these effects could not be replicated in human studies or clinical trials due to bioavailability limitations. Given the high content of stilbene-based derivatives in *Vaccinium* berries [27,28], it is possible to consider them potential therapeutic agents in BC. However, these derivatives are also found in grapes and wine [74]. While this indicates that these derivatives have therapeutic effects, it is unclear if these effects are significant enough to make them useful for BC treatment. The identification and extraction of anthocyanins from *Vaccinium* berries in a few experiments are unknown.

The few in vivo experiments that have been conducted show that *Vaccinium* berries and their bioactives have therapeutic potential against BC cells. Despite remarkable success in rodent models, these models are limited in their capability to accurately mimic the efficacy of cancer treatment and adverse treatment events [75]. Thus, the therapeutic efficacy of *Vaccinium* berries and their bioactives in BC identified in animal models could not be translated to clinical trials.

The composition profiles of *Vaccinium* berries were not similar in the majority of experiments. The levels and composition of natural bioactives vary depending on their species and storage conditions. The anthocyanin and phenolic acid content of blueberry and bilberry are much higher than in cranberry. Thus, the content and levels of each bioactive compound in *Vaccinium* berries are highly different. 

The mechanisms by which *Vaccinium* bioactives are thought to be effective in BC treatment have not been clearly described. Although cranberry and/or bilberry-derived flavanols have significant anti-BC activities, the mechanisms underlying their effect are not explored. The extraction of *Vaccinium* bioactives differed across the summarized experiments, making it difficult to compare results between in vitro and in vivo models. A few experiments have been conducted following treatment of BC cells with blueberry and cranberry extracts without clearly defining the extraction of these berries. Experiments utilizing cranberry, bilberry, and their bioactives showed lower BC inhibitory mechanisms than those using blueberry, making them the least effective of the *Vaccinium* berries against BC cells. The majority of experiments have not been reported to evaluate the potential safety and toxicity of *Vaccinium* bioactives in BC cells. Experimental models have shown inhibitory effects of *Vaccinium* bioactives against BC cells, but the optimal effective dose in treatment remains unknown.

## 6. Concluding Remarks

*Vaccinium* spp. are fleshy berry fruits that contain various levels of bioactives depending on their species, ripeness stage, and growth location. The findings summarized suggest that *Vaccinium* berries and their bioactives may have efficacy as anti-BC agents. Blueberry, cranberry, bilberry, and lingonberry have shown promising results in BC treatment. These berries and their bioactives (flavanols, phenolic acids, and stilbene-based derivatives) have exerted therapeutic effects against BC cells, demonstrated by the inhibition and/or activation of the cellular signaling pathways/molecular genes involved in estrogen-induced mammary tumorigenesis, mammosphere formation, inflammation, proliferation, metastasis, migration, invasion, viability, adhesion, and anti-apoptosis/autophagy. The combination treatment of *Vaccinium* bioactives (anthocyanins, pterostilbene, and resveratrol) and chemotherapeutic drugs has been shown to exert anti-proliferative and apoptotic/autophagic effects in BC cells. 

## 7. Future Directions

*Vaccinium* berries and their bioactives are promising therapeutic agents for BC in experimental models. Data gathered from experimental models should be evaluated rigorously and provide evidence of treatment effectiveness before even going to human clinical trials. Clinical trials are needed to extend the evidence that the bioactive compounds of *Vaccinium* berries can be used as medicinal properties with therapeutic potential in BC. Despite stilbene-based derivatives being proven to be effective in multiple experimental models, there is still need for clinical trials and human studies on their efficacy in targeting BC. Further clinical trials testing durations of treatment, dosages, and the safety/toxicity of different *Vaccinium* berries and their bioactives in BC patients are also needed. A more precise characterization of the unknown anthocyanins present in *Vaccinium* berry extracts is needed in future studies. 

*Vaccinium* bioactives may have different mechanisms of action for the treatment of BC both in vitro and in vivo, and these mechanisms require further investigation. Further studies are required to elaborate the mechanisms underlying the therapeutic potential of bioactives derived from other *Vaccinium* berries (e.g., bog bilberry) against BC cells. Additional experiments are needed to understand the mechanisms of *Vaccinium* bioactives in combination with chemotherapeutic drugs in BC treatment.

## Figures and Tables

**Table 1:** The effects and mechanisms of action of blueberry and cranberry in BC treatment.

Study Type	Model (Cell Line)	*Vaccinium* Berries	Treatment	Effects on BC	Mechanisms of Action	Refs.
In vitro	MCF7 and T47D	Blueberry	Blueberry was extracted with 50% hexane/ethyl acetate, ethyl acetate, ethanol, and 70% acetone/water; evaporated at 40 °C; and then incubated for 48 h	Inhibition of carcinogen benzo(a)pyrene-mediated mutagenesis	Metabolically activated and direct-acting carcinogen methyl methanesulfonate	[42]
In vitro/vivo	HCC38, HCC1937, MCF10A, and MDA-MB-231	Blueberry	BALB/c Nu-Nu athymic female mice were fed daily with 100 μL/kg BW blueberry extract for 6 weeks BC cells were treated with blueberry extract at 0, 5, 10, 20, 40, and 80 μL/mL concentrations and incubated for 72 h	Inhibition of cell proliferation, migration, and motility Induction of cell apoptosis	PAI, TIMP, caspase-3 ↑ uPA, MMP-2, MMP-9, HGF, Ki-67, PI3K, Akt, NFκB ↓	[43]
In vivo	MDA-MB-231	Blueberry	BALB/c Nu-Nu athymic female mice were fed with 5% and 10%/kg BW blueberry powder for 6 weeks	Inhibition of cell proliferation and metastasis Induction of cell apoptosis Increased levels of anti-inflammatory cytokines	Caspase-3, IL-13, IFNα, APC ↑ Ki-67, GSK-3β, β-catenin ↓	[44]
In vivo	MDA-MB-231-luc-D3H2LN	Blueberry	BALB/c Nu-Nu athymic female mice were fed with 5%/kg BW wholeblueberry powder for 15 weeks	Inhibition of tumor growth and metastasis Increased levels of anti-inflammatory cytokines Decreased levels of pro-inflammatory cytokines	IFNα, IP-10, IL-12, IL-2 ↑ IL-17, IL-10, IL-4, VEGF, MIP-1α ↓	[45]
In vivo	NA	Blueberry	Female ACI rats received 5%/kg BW blueberry powder (Tifblue and Rubel) and E2 treatment 2 weeks prior to treatment and after 12 weeks	Inhibition of cell proliferation	Cytochrome CYP 1A1, ER-α, cyclin D1, PCNA, miR-18a, miR-34c ↓	[46]
In vivo	NA	Blueberry	Female ACI rats received 2.5%/kg BW blueberry and E2 treatment over 24 weeks	Inhibition of estrogen-induced mammary tumorigenesis	CYP1A1, COMT ↓	[47]
In vitro/vivo/ex vivo	4T1, MDAMB-231, and MCF7	Blueberry	BALB/c female mice received either NBJ or PEBP at 12.5, 25, and 50% (kg BW) concentrations for 2 weeks BC cells were treated with NBJ and PEBP at 150 or 200 μM concentrations and incubated for 24 h	Inhibition of cell proliferation, tumor growth, metastasis, invasion, mobility, and mammosphere formation	IL-6, MAPK p38, PTEN, JNK/SAPK ↑ STAT3 PI3K/Akt, ERK 1/2 ↓	[48]
In vitro	4T1 and MB-MDM-231	Blueberry	BC cells were treated with NBJ and PEBP at 40, 60, 100, 150, and 200 μM concentrations and incubated for 24 h	Inhibition of cell invasion BC chemoprevention	miR-145, FOXO1 ↑ miR-210, NRAS ↓	[49]
In vitro	MDA-MB-231	Blueberry, cranberry	BC cells were treated with blueberry and cranberry at 0, 10, 20, 30, 40, and 50 μL/mL concentrations and incubated for 48 h	Inhibition of cell proliferation Induction of cell cycle arrest	cdk4/6, cyclin D1/D3, TNF, COX-2, NFκB ↓	[50]

(↓) Decrease, (↑) increase; NA = not available.

**Table 2:** The effects and mechanisms of action of flavanols and phenolic acids in BC treatment.

Study Type	Model (Cell Line)	*Vaccinium* Berries	Bioactive Compounds	Treatment	Effects on BC	Mechanisms of Action	Refs.
In vitro	MDA-MB-435 and MCF7	Cranberry	Flavanols (anthocyanins not specified, proanthocyanidins, and catechins)	BC cells were treated with cranberry press cake containing flavanol compounds at 0–200 μg/mL concentrations and incubated for 24 h	Inhibition of cell proliferation Induction of apoptosis and cell cycle arrest	NA	[51]
In vitro	MCF7	Cranberry	Flavanols (anthocyanins, cyanidin, and catechins), gallic acid	BC cells were treated with cranberry phytochemical extracts at 0–60 μg/mL concentrations and incubated for 24 h	Inhibition of cell proliferation Induction of apoptosis and cell cycle arrest	NA	[52]
In vitro	MCF7	Blueberry, cranberry	Anthocyanins (malvidin, peonidin, petunidin, delphinidin, and cyanidin)	BC cells were treated with blueberry and cranberry at 25, 50, 100, 150, and 200 μg/mL concentrations and incubated for 24 h	Inhibition of cell proliferation	NA	[53]
In vitro	MCF7 and MDAMB-231	Blueberry	Anthocyanins (not specified)	BC cells were treated with extract I (anthocyanin from blueberry) and extract II (anthocyanin-pyruvic acid adduct) at 50, 100, 250, and 500 μg/mL concentrations and incubated for 24 h	Inhibition of cell proliferation and invasion Induction of cell apoptosis	Caspase-3 ↑	[54]
In vivo	T47D	Blueberry	Anthocyanins (malvidin, peonidin, petunidin, delphinidin, and cyanidin)	Female rats were fed with 5%/kg BW blueberry powder. BC cells were treated with blueberry powder at 25, 50, 100, 200, or 400 μM concentrations and evaluated after 75–200 days	Inhibition of cell proliferation	CYP1A1 ↓	[55]
In vivo	MCF7	Blueberry	Anthocyanins (not specified)	Administration of gardenblue blueberry anthocyanins to BALB/c nude mice via an intravenous injection (dose = 10 mg/kg) over 25 days	Inhibition of cell proliferation Induction of cell apoptosis	DNA damage ↓	[56]
In vivo/ex vivo	4T1 and MB-MDM-231	Blueberry	Protocatechuic acid, gallic acid, and catechol	BALB/c female mice were fed with a polyphenolic mixture comprising protocatechuic acid (70 mg/kg BW), gallic acid (35 mg/kg Bw), and catechin (1.5 mg/kg Bw) BC cells were treated with a polyphenolic mixture containing protocatechuic acid, gallic acid, and catechin at 1 and 2 μM concentrations and incubated for 24 h	Inhibition of mammosphere formation in BC cells	miR-145, FOXO1 ↑	[57]
In vivo/ex vivo	MCF7 and MDA-MB-231	Blueberry	Phenolic acids/hippuric acid	Wild-type female and male mice were fed with a 10%/kg BW blueberry phenolic acid mixture The effect of hippuric acid on mammosphere formation was evaluated at 3 μg/mL and 10 μg/mL/kg BW after 5 days	Inhibition of mammosphere formation in BC cells	PTEN ↑	[58]
In vitro	MCF7-GFP tubulin	Bilberry	Anthocyanins (not specified)	BC cells were treated with bilberry anthocyanin extract at 0.125, 0.25, 0.5, and 1.0 mg/mL and incubated for 72 h	Inhibition of microtubule polymerization and cell proliferation Induction of apoptosis and cell cycle arrest	NA	[59]
In vitro/vivo	MDA-MB-231 and MCF7	Bilberry	Anthocyanidin aglycone (Anthos)	Female athymic nu/nu mice were randomized into Anthos (10 mg/kg Bw), ExoAnthos (5 mg Anthos and 50 mg Exo protein/kg Bw), and vehicle (PBS) BC cells were treated with Anthos and ExoAnthos at 0–100 μM concentrations and incubated for 24 h	Inhibition of cell proliferation and inflammation	TNFα, NF-κB ↓	[60]
In vitro/vivo	MDA-MB-231, MDA-MB-236, and HCC1937	Bilberry	Anthocyanidin aglycone (Anthos)	Athymic nude/NOD-Scid mice were randomized into vehicle (PBS) and Anthos (30 mg/kg and 60 mg/kg Bw three times a week) BC cells were treated with Anthos at 0–200 μM concentrations and incubated for 24–72 h	Inhibition of cell proliferation, viability, migration, invasion, and metastasis Induction of apoptosis and cell cycle arrest	Cleaved caspase-3/7/9, cleaved PARP, E-cadherin, β-actin ↑ NF-kB, IkB Kinase, TNFα, N-cadherin, vimentin, snail, slug, cyclin A/B1/E2 ↓	[61]
In vitro	MCF7	Bilberry	Flavanols (procyanidin, catechin, anthocyanins malvidin, peonidin, petunidin, delphinidin, and cyanidin), phenolic acids (gallic, quinic, caffeoylquinic, and caffeic)	BC cells were treated with 50 μL bilberry extracts at 0.125, 0.25, and 0.5 mg DW/mL concentrations and incubated for 24 h	Inhibition growth of pathogenic strains	NA	[62]

(↓) Decrease, (↑) increase; NA = not available.

**Table 3:** The effects and mechanisms of action of stilbene-based derivatives in BC treatment.

Study Type	Model (Cell Line)	*Vaccinium* Berries	Bioactive Compounds	Treatment	Effects on BC	Mechanisms of Action	Refs.
In vitro	MDA-MB-231 and MCF7	Blueberry	Pterostilbene	BC cells were treated with pterostilbene at 25 or 75 μM concentrations and incubated for 24 h	Inhibition of cell proliferation and viability Induction of apoptosis and cell cycle arrest	Caspase-3/7, superoxide anion ↑	[63]
In vitro	MCF7, ZR-751, and MDA-MB-231	Blueberry	Pterostilbene	BC cells treated with pterostilbene (10, 20, or 30 μmol/L) and tamoxifen (5 μmol/L) for 72 h	Inhibition of cell viability Induction of apoptosis	NA	[64]
In vitro	MCF7	Blueberry	Pterostilbene	BC cells were treated with pterostilbene at 0, 5, 10, 20, and 30 μM concentrations and incubated for 24 h	Inhibition of cell proliferation, viability, and invasion Inhibition of colony formation and wound healing in BC cells	MMP-9, PI3K-Akt, p38 kinase ↓	[65]
In vitro/vivo	MCF7 and MDA-MB-231	Blueberry	Pterostilbene	Female nude mice were administered with 56 mg/kg pterostilbene once every four days for 3 weeks BC cells were treated with pterostilbene at 7.5, 15, and 30 μM concentrations and incubated for 72 h	Inhibition of cell proliferation Induction of cell apoptosis	ER-α36, MAPK/ERK, PI3K/Akt ↓	[66]
In vitro	MDA-MB-231/468, MCF7, and BT-20	Blueberry	Pterostilbene	BC cells were treated with pterostilbene at 10, 20, 40, and 80 μM concentrations and incubated for 12, 24, and 48 h	Inhibition of viability and colony formation in BC cells Induction of cell apoptosis	Caspase-3/8/9, PARP, DR 4/5, Bax 67, ROS ↑ DcR-1/2, Bcl-2, Bcl-XI, Survivin, c-FLIPS/L, XIAP ↓	[67]
In vitro/vivo	MCF7 and MDA-MB-231	Blueberry	Pterostilbene	NOD/SCID mice were injected with 5 mg/kg pterostilbene dissolved in corn oil five times/week BC cells were treated with pterostilbene at 2.5, 5, and 10 μM concentrations and incubated for 48 h	Inhibition of cell migration/metastasis and invasion Inhibition of tumor sphere formation ability	E-cadherin, β-actin, miR-448 ↑ Twist1, vimentin, β-catenin, HIF-1α, NFκB ↓	[68]
In vitro/vivo	MCF7, SK-BR-3, and MDA-MB-468	Blueberry	Pterostilbene	Female nude mice were administered with 0.1% pterostilbene weekly over 8 weeks BC cells were treated with pterostilbene at 0, 25, 50, 75, and 100 μM concentrations and incubated for 72 h	Inhibition of cell proliferation Induction of apoptosis and cell cycle arrest	Bax, p21, ERK 1/2 ↑ AKT, mTOR, cyclin D1 ↓	[69]
In vitro	MCF7 and MDA-MB-231	Blueberry	Pterostilbene	BC cells were treated with pterostilbene at 0, 5, 7.5, and 10 μM concentrations and incubated for 4 h	Inhibition of cell proliferation Induction of apoptosis and cell cycle arrest	hTERT, cMyc, telomerase ↓	[70]
In vitro	MDA-MB-231	Blueberry	Piceatannol	BC cells were treated with piceatannol at 2, 5, and 10 μM concentrations and incubated for 24 h	Inhibition of cell migration, invasion, and adhesion	PTEN ↑ MMP-9, PI3K, Akt, mTOR, NF-κB, IκBα ↓	[71]
In vitro/vivo	MDA-MB-435s, MDA-MB-231, and SKBR-3	Blueberry, bilberry, cranberry, and lingonberry	Resveratrol	Female athymic nu/nu mice were injected with 10 mg/kg Bw paclitaxel and 16.5 mg/kg Bw resveratrol three times a week BC cells were treated with 10 nM paclitaxel with or without 20 μM resveratrol and incubated for 48 h	Inhibition of cell viability Induction of apoptosis and cell cycle arrest	Chk2 ↑ Bcl-xL, Bcl-2, PARP, ROS ↓	[72]
In vitro	SKBR-3	Blueberry, bilberry, cranberry, and lingonberry	Resveratrol	BC cells were treated with resveratrol at 0–150 μM concentrations and incubated for 24, 48, and 72 h	Inhibition of cell viability Induction of apoptosis and cell cycle arrest	PEA3, PTEN ↑ FASN, cyclin D1, Akt ↓	[73]

(↓) Decrease, (↑) increase; NA = not available.

## Data Availability

Not applicable.

## Abbreviations

ACI	August-Copenhagen-Irish
Akt	Threonine kinase
APC	Adenomatous polyposis coli
Bax	Bcl-2-associated X protein
BC	Breast cancer
Bcl-2	B-cell lymphoma-2
Bcl-xl	B-cell lymphoma-extra large
BL	Basal-like
CDK	Cyclin-dependent kinase
Chk2	Checkpoint kinase 2
CHOP	p38/C/EBP-homologous protein
COMT	Catechol-O-methyl transferase
COX-2	Cyclooxygenase-2
CSCs	Cancer stem cells
CYP1A1	Cytochrome P4501A1
DcR	Decoy receptor
DR	Death receptor
E2	Estradiol
EMT	Epithelial-to-mesenchymal transition
ER	Estrogen
ERK	Extracellular-signal regulated kinase
FASN	Fatty acid synthase
FOXO1	Forkhead box O1
GSK	Glycogen synthase kinase
HER2	Human epidermal growth factor receptor-2
HGF	Hepatocyte growth factor
HIF-1 α	Hypoxia-inducible factor 1-α
HRG-β1	Heregulin-β1
hTERT	Human telomerase reverse transcriptase
IFNα	Interferon-α
IL	Interleukin
IM	Immunomodulatory
IκBα	NF-κB alpha
JNK	c-Jun N-terminal kinase
LAR	Luminal androgen receptor
M	Mesenchymal
MAPK	Mitogen-activated protein kinase
MIG	Monokine induced by interferon- γ
MIP-1α	Macrophage inflammatory protein-1α
miR	microRNA
MMP	Matrix mettaloproteinase
MMP	Matrix metalloproteinase
MSL	Mesenchymal stem-like
mTOR	Mammalian target of rapamycin
NBJ	Non-fermented blueberry juice
NET	Neoadjuvant endocrine therapy
NFκB	Nuclear factor kappa-B
NRAS	Neuroblastoma RAS Viral Oncogene Homolog
PAI	Plasminogen activator inhibitor
PARP	Poly (ADP-ribose) polymerase
PCNA	Proliferating cell nuclear antigen
PEA3	Polyomavirus enhancer activator 3
PEBP	Polyphenol-enriched blueberry preparation
PI3K	Phosphatidylinositol 3-kinase
PR	Progesterone
PTEN	Phosphatase and tensin homolog
ROS	Reactive oxygen species
SAPK	Stress-activated protein kinase
STAT3	Signal transducer and activator of transcription 3
TAMs	Tumor-associated macrophages
TIMPs	Tissue inhibitors of metalloproteases
TNBC	Triple-negative breast cancer
TNF	Tumor necrosis factor
TRAIL	Tumor necrosis factor-related apoptosis-induced ligand
uPA	Serine protease
VEGF	Vascular endothelial growth factor
